# Application of Rho Kinase Inhibitors for the Treatment of Corneal Endothelial Diseases

**DOI:** 10.1155/2017/2646904

**Published:** 2017-07-02

**Authors:** Naoki Okumura, Shigeru Kinoshita, Noriko Koizumi

**Affiliations:** ^1^Department of Biomedical Engineering, Faculty of Life and Medical Sciences, Doshisha University, Kyotanabe, Japan; ^2^Department of Frontier Medical Science and Technology for Ophthalmology, Kyoto Prefectural University of Medicine, Kyoto, Japan

## Abstract

ROCK (Rho kinase) signaling regulates a wide spectrum of fundamental cellular events and is involved in a variety of pathological conditions. It has therefore attracted research interest as a potential therapeutic target for combating various diseases. We showed that inhibition of ROCK enhances cell proliferation, promotes cell adhesion onto a substrate, and suppresses apoptosis of corneal endothelial cells (CECs). In addition, we reported that a ROCK inhibitor enhances wound healing in the corneal endothelium in animal models and in pilot clinical research. We also demonstrated the usefulness of a ROCK inhibitor as an adjunct drug in tissue engineering therapy as it enhances the engraftment of CECs onto recipient corneas. In 2013, we initiated a clinical trial to test the effectiveness of injection of cultured human CECs into the anterior chamber of patients with corneal endothelial decompensation. This paper reviews the accumulating evidence supporting the potency of ROCK inhibitors in clinical use, both as eye drops and as adjunct drugs in cell-based therapies, for the treatment of corneal endothelial decompensation.

## 1. Introduction

The corneal endothelium, through its pump-and-leak barrier functions, maintains corneal transparency by regulating the amount of water inside the corneal stroma. One clinical feature of the corneal endothelial cell (CEC) phenotype is poor regenerative ability, as CECs have severely limited proliferative capacity [1]. Consequently, any damage to the corneal endothelium is repaired by compensatory migration and spreading of the residual CECs to cover the wounded area, with a resulting drop in the CEC density. This density is typically 2000–2500 cells/mm^2^ in a normal subject, and a drop below a critical level, usually less than 500–1000 cells/mm^2^, can result in a hazy cornea due to decompensation of the corneal epithelium.

The only current therapeutic choice for treating corneal endothelial decompensation is corneal transplantation using donor corneas [2]. Penetrating keratoplasty, in which a whole-thickness cornea is replaced with a donor cornea, has been performed since 1906 [3]. Descemet's stripping endothelial keratoplasty (DSEK) was introduced in the clinical setting in the last decade to reduce the invasiveness of penetrating keratoplasty and to improve clinical outcomes and is now performed routinely worldwide, largely replacing penetrating keratoplasty. The further introduction and gradual adoption of Descemet's membrane endothelial keratoplasty (DMEK) is now resulting in higher recovery of visual quality even in comparison to DSEK [4].

**Figure 1:**
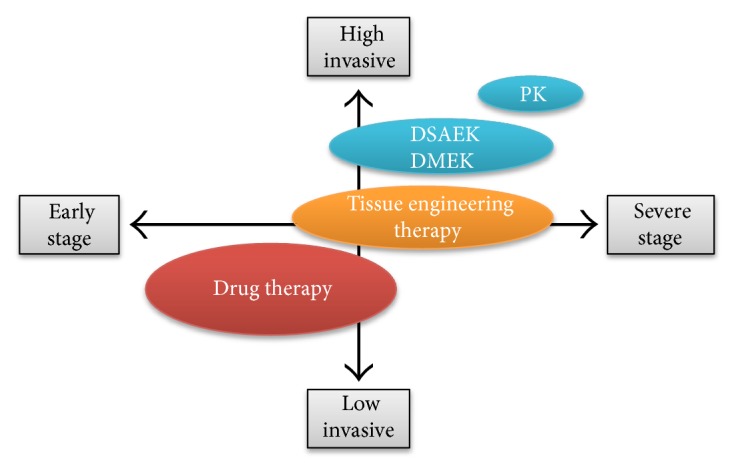
Future treatment strategies for corneal endothelial decompensation. Pharmaceutical treatments and tissue engineering therapies are possible innovative therapeutic modalities. Reproduced from Okumura [49].

The evolution of surgical procedures has enabled less invasive treatment of corneal endothelial decompensation with better clinical outcomes. However, these surgeries still have associated issues, such as the difficulty of the actual surgical technique, graft rejection, acute and chronic cell loss, and the shortage of donor corneas. Therefore, new and innovative therapies are still in great demand. One research direction focuses on tissue engineering therapy, and another is pharmaceutical treatment (Figure 1). We have proposed the use of Rho kinase (ROCK) inhibitors for both pharmaceutical and tissue engineering treatments, and our preliminary results in clinical research indicate success for both applications. This review provides an overview of the research into ROCK inhibitors and their potential for the clinical treatment of corneal endothelial decompensation.

## 2. ROCK Signaling as a Potent Therapeutic Target for Various Diseases

Rho is a small GTPase that is activated by guanine nucleotide exchange factors (GEFs). Upon binding to GTP, RhoA activates ROCK, a serine/threonine kinase that phosphorylates various substrates. ROCK, initially isolated as a GTP-bound protein, has two isoforms, ROCK I and ROCK II, which share a 60% similarity in amino acid sequence and a 90% similarity in the kinase domain [5, 6]. ROCK signaling regulates a wide spectrum of fundamental cellular events, such as cell adhesion, motility, proliferation, differentiation, and apoptosis [6, 7]. ROCK signaling is involved in variety of diseases, including vascular disease, cancer, asthma, insulin resistance, kidney failure, osteoporosis, neuronal degenerative disease, and glaucoma [8–10]. ROCK signaling has therefore attracted interest as a potential therapeutic target for these diseases.

Two ROCK inhibitors have been approved for use in the clinical setting. Fasudil was approved in 1995 in Japan, where it is used to suppress cerebral vasospasm by inhibition of actomyosin contraction [9]. Ripasudil was approved in Japan in 2014 in an eye drop form to increase the outflow of the aqueous humor as a treatment for glaucoma and ocular hypertension [11].

The importance of ROCK signaling is generally accepted in a wide spectrum of cellular events and pathological conditions; however, its role varies depending on cell type and cell status [12]. In 2009, we found that the use of a selective ROCK inhibitor, Y-27632, enhanced CEC proliferation, promoted CEC adhesion onto a substrate, and suppressed CEC apoptosis [13]. These findings confirmed that the use of a ROCK inhibitor could greatly improve our ability to culture CECs for subsequent application in tissue engineering therapies. However, we quickly discovered that the benefits of ROCK inhibitor use were not limited only to efficient cell cultivation.

## 3. ROCK Inhibitors Can Promote Wound Healing in the Corneal Endothelium

Early studies showed that inactivation of Rho blocks serum-stimulated DNA synthesis, whereas activation of RhoA induces G_1_/S progression in 3T3 fibroblasts [14, 15]. A majority of subsequent studies confirmed that inhibition of the Rho/ROCK signaling pathway suppresses cell cycle progression in various types of cells, including lung carcinoma, melanoma, and kidney tumor cells [16–18]. However, our analysis showed that treatment of CECs with a ROCK inhibitor increased cyclin D levels and suppressed phosphorylation of p27^kip1^ by activation of phosphatidylinositol 3-kinase signaling [19]. Cyclin D and p27 are regulators of the G_1_/S progression, so this explained how ROCK inhibition promoted the proliferation of CECs [19]. Subsequent researchers also demonstrated that the use of the ROCK inhibitor Y-27632 increased cell proliferation [20], although one study found no enhanced CEC proliferation following Y-27632 treatment [21]. Peh and colleagues explained this discrepancy by suggesting that CECs derived from young donors are more responsive to Y-27632 and the effect of ROCK inhibition can vary depending on the status of the cornea [20]. More studies are needed to clarify the role of corneal status on the response to ROCK inhibitors and promotion of CEC proliferation.

## 4. ROCK Inhibitor Eye Drops Promote Wound Healing in the Corneal Endothelium of Animal Models

The administration of a ROCK inhibitor in an eye drop form promoted corneal endothelial wound healing in a rabbit model in which the central corneal endothelium was damaged with a stainless steel cryoprobe (6 mm diameter) [19, 22]. Two ROCK inhibitors, Y-27632 and Y-39983, enhanced the rate of wound healing and accelerated the recovery of corneal transparency. The numbers of Ki67-positive proliferating cells were also increased by administration of the eye drops, suggesting that ROCK inhibition promoted CEC proliferation both in vitro and in vivo. However, the rabbit corneal endothelium has proliferative ability, whereas this ability is limited in the primate model and in humans [23–25]. We confirmed that ROCK inhibitor eye drops promoted similar corneal endothelial wound healing in the primate animal model to that observed in the rabbit model [26].

**Figure 2:**
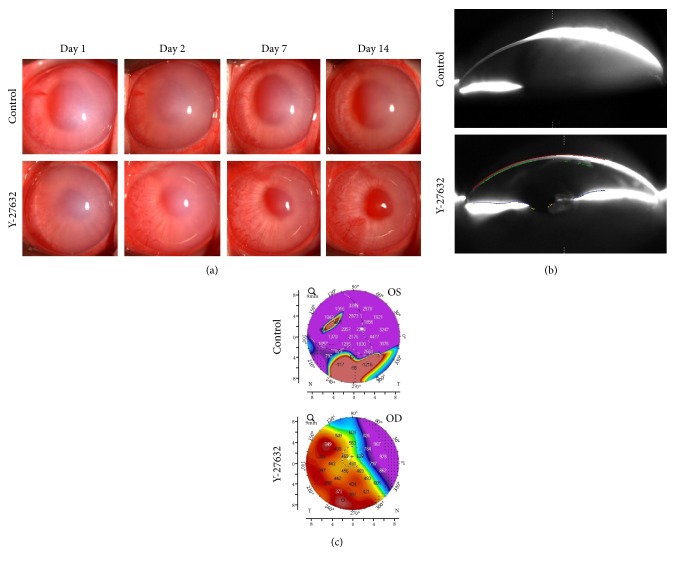
Effect of Y-27632 on wound healing in a corneal endothelial damage rabbit model. (a) Half of the corneal endothelium area was mechanically scraped. Y-27632 (10 mM) diluted in phosphate-buffered saline (PBS) was applied topically 6 times daily, and PBS was applied 6 times daily as a control. Corneal transparency was assessed by slit-lamp microscopy for 14 days (*n* = 6). Representative anterior segment images are shown. (b, c) Anterior segments were also evaluated with a Pentacam®. Representative Scheimpflug images and corneal thickness maps obtained with the Pentacam HR are shown. Values in the corneal thickness map are indicated in *μ*m. Reproduced from Okumura et al. [27].

The cryoprobe damaged both the corneal epithelium and the corneal endothelium, so we used a different rabbit model, in which a semicircular area accounting for 50% of the corneal endothelium was scraped from the Descemet's membrane with a 20-gauge silicone needle [27, 28]. The use of ROCK inhibitor eye drops in this surgical model also resulted in enhanced wound healing and a significant increase in Ki67-positive proliferating cells when compared with the use of the vehicle in control eyes. Five out of 6 control eyes exhibited hazy corneas due to corneal endothelial decompensation after 2 weeks, but 6 out of 6 eyes treated with Y-27632 drops exhibited transparent corneas (Figure 2).

## 5. Are ROCK Inhibitor Eye Drops Clinically Useful for the Treatment of Fuchs Endothelial Corneal Dystrophy?

**Figure 3:**
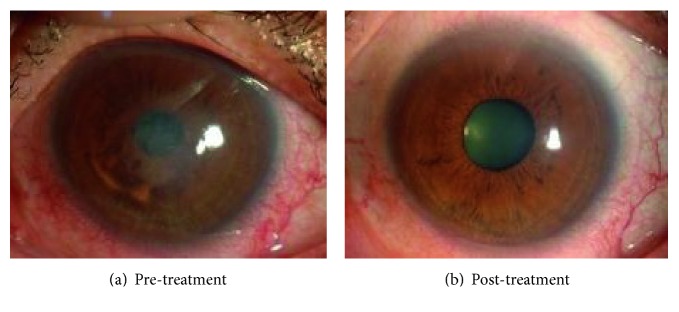
Clinical research on the use of ROCK-inhibitor Y-27632 eye drops for treatment of corneal decompensation. A representative case of a patient with central corneal edema due to Fuchs endothelial corneal dystrophy is shown. Before treatment, central corneal edema was observed (a), but the corneal edema was eliminated and visual acuity recovered from logMAR 0.70 to −0.18 after 6 months of treatment (b). Reproduced from Okumura et al. [26].

We obtained approval from the institutional review board of the Kyoto Prefectural University of Medicine in 2010 to test ROCK inhibitor eye drops as a treatment for corneal endothelial dysfunction (approval number C-626-2) [26, 29]. Eight patients with corneal endothelial dysfunction who were scheduled for DSEK were enrolled in our clinical research study. Patients were categorized into 2 groups: (1) a central edema group, whose corneal edema was evident in the corneal center and who had a relatively transparent area remaining in the peripheral area (all 4 patients in this group had diagnoses of early- to intermediate-stage Fuchs endothelial corneal dystrophy), and (2) a diffuse edema group, whose corneal edema was observed throughout the cornea, from the center to the periphery. The central corneal endothelium was removed with a 2 mm diameter stainless cryoprobe, and the eyes were then treated with Y-27632 eye drops (10 mM) applied 6 times daily for 7 days. The limited number of patients precluded obtaining statistically significant differences, but a clear trend was observed for a reduction in central corneal thickness in the central edema group, but not in the diffuse edema group, in response to the eye drop treatment. Notably, one patient in the central edema group, a 52-year-old Japanese male diagnosed with late-onset Fuchs endothelial corneal dystrophy, showed a dramatic recovery. His pretreatment central corneal thickness of 703 *μ*m and visual acuity of 20/63 recovered to 568 *μ*m and 20/20, respectively, after the ROCK inhibitor treatment (Figure 3). Contact specular microscopy showed that the average corneal endothelial densities in the central and peripheral cornea were 1549 ± 90 and 705 ± 61 cells/mm^2^, respectively, after 18 months. We were unable to perform specular microscopy in the central cornea because of the edema that existed before the treatment, but mild guttae were detected, mainly from the central to the paracentral area, after treatment [26, 29]. The limitation of this pilot clinical research was the small number of the patients and the absence of control cases who underwent the same central corneal endothelial removal with the cryoprobe but not with the ROCK inhibitor treatment. In addition, transcorneal freezing with a cryoprobe probably removes only corneal endothelial cells but not Descemet's membrane with guttae. A further study is necessary to determine whether corneal endothelial cells repopulate on the guttae and retain a functional status or whether the cryoprobe affects the guttae. The inflammatory response due to damaged epithelial cells and keratocytes can affect the corneal endothelial wound healing process. Accordingly, several other research groups have recently tested the effect of ROCK inhibitor eye drops for the treatment of early-stage Fuchs endothelial corneal dystrophy following Descemet's membrane removal instead of cryoprobe removal of the central corneal endothelium, but the clinical response seems to be controversial [30–32]. Further randomized clinical trials in larger cohorts are necessary to validate the effectiveness of ROCK inhibitor eye drops as a treatment for Fuchs endothelial corneal dystrophy.

## 6. Are ROCK Inhibitor Eye Drops Effective at Preventing Postcataract Surgery Corneal Decompensation?

Another possible indication for ROCK inhibitor administration is acute corneal endothelial damage, especially that occurring during cataract surgery. Corneal decompensation following cataract surgery is one of the leading causes of corneal transplantation in many countries, accounting for 12.2% and 20–40% of the corneal transplantations performed in the United States and in Asian countries, respectively [33–35]. The factor that determines whether the cornea becomes transparent or undergoes corneal decompensation is the density of CECs available for redistribution. Corneal haze will occur if the CEC density is insufficient. We hypothesized that ROCK inhibitor eye drops could in fact promote the proliferation of the residual CECs following corneal endothelial damage and increase the numbers of CECs available for coverage, thereby reducing the risk of corneal decompensation [36].

**Figure 4:**
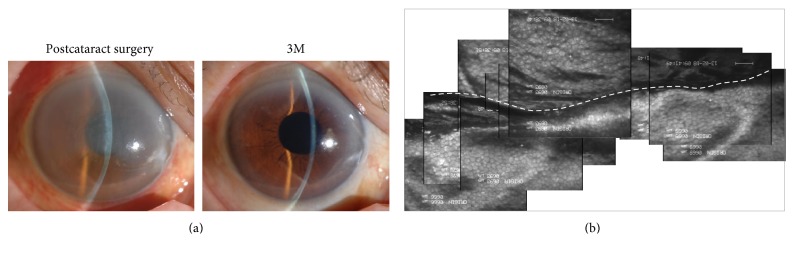
Pilot clinical research on the use of ROCK inhibitor eye drops for the treatment of acute corneal endothelial damage due to cataract surgery. (a) An 84-year-old female diagnosed with cataract underwent phacoemulsification. During the surgery, the Descemet's membrane was spontaneously detached from the upper incision tunnel and over 2/3 was aspirated. The patient was referred to the cornea clinic of Kyoto Prefectural University of Medicine due to severe corneal edema. The patient was treated with the 1 mM Y-27632 eye drops for 6 months. At 2 weeks, the cornea had recovered its clarity, and the patient's visual acuity had improved to 20/20 at 3 months. (b) Panoramic image of the corneal endothelium, evaluated by contact specular microscopy after 3 months. The Descemet's removal line is indicated as a white dotted line. Reproduced from Okumura et al. [27].

Our findings are preliminary, but our evaluation of the safety and effectiveness of ROCK inhibitor eye drops supports their use as a treatment for corneal endothelial damage [27]. Two patients were referred to the cornea clinic in Kyoto Prefectural University of Medicine after more than half of the area of the Descemet's membrane was accidentally removed during cataract surgery. One additional patient had undergone cataract surgery and removal of an iris cyst that had formed following a previous eye trauma, and almost half of the area of the cornea was exposed where the iris cyst was removed. All three patients showed severe corneal edema after surgery and were at high risk for subsequent corneal decompensation. However, the administration of ROCK inhibitor eye drops led to the recovery of corneal transparency within 1-2 months in all three eyes (Figure 4). Recovery time tends to be faster than previously reported, where patients with iatrogenic Descemet's membrane removal during cataract surgery spontaneously recovered corneal clarity within 1–6 months [37–39]. However, further randomized clinical trials are needed to validate these findings and to confirm the effectiveness of ROCK inhibitors as a treatment for corneal endothelial damage. However, the positive findings from these preliminary clinical cases have motivated us to develop ROCK inhibitors as drugs for the treatment of corneal endothelial damage due to cataract surgery.

Ripasudil eye drops were approved in Japan as GLANATEC® ophthalmic solution 0.4% for the treatment of glaucoma and ocular hypertension [11]. In rabbit experiments, we demonstrated that ripasudil shows similar effects on corneal endothelial wound healing as other ROCK inhibitors [28]. Hence, repositioning of ripasudil as a drug for corneal endothelial diseases seems to be one possible strategy to bring ROCK inhibitors quickly into clinical settings.

## 7. ROCK Inhibitor Treatment Enables Cell-Based Therapy

Tissue engineering has been anticipated as new therapy that can replace conventional corneal transplantation using donor corneas. At least two possible strategies are available for transplanting cultured CECs to recipient corneas [40]. One strategy is to make a cultured corneal endothelial sheet and transplant it in a procedure much like DSEK or DMEK. We and several other researchers have cultured CECs on a substrate and transplanted the resulting CEC sheet into animal models [41–43]. The transplanted CEC sheet regenerated a transparent cornea in animal models, but the transplantation of the fragile monolayer sheet into the anterior chamber and attachment onto the back side of the cornea involve difficult surgical techniques.

The second strategy is to inject cultured CECs in the form of a cell suspension into the anterior chamber. The CECs injected into the anterior chamber will not spontaneously attach to the recipient corneal epithelial layer, so magnetic guidance or injection of CEC spheres was proposed to enhance the CEC engraftment [44–46]. We considered cell injection to have several advantages over sheet transplantation, including a simplified transplantation procedure, less invasiveness to patients, easier preparation of cell stock, and avoidance of artificial substrate use. Our finding that ROCK inhibitor treatment enhances adhesion of CECs onto a substrate [13] prompted us to initiate animal experiments in which cultured CECs were injected into the anterior chamber in combination with a ROCK inhibitor. We used two animal models of corneal endothelial dysfunction (rabbit and cynomolgus monkey), injected cultured CECs in the form of a cell suspension combined with a ROCK inhibitor, and demonstrated regeneration of the corneal endothelium and restoration of a transparent cornea [47, 48]. We have since accumulated evidence that confirms the safety and effectiveness of cultured CEC injections in combination with a ROCK inhibitor in animal models. The safety and functional profiles of cultured human CECs for clinical use have also been carefully evaluated [48].

## 8. Clinical Study of Cell-Based Therapy Using ROCK Inhibitors

**Figure 5:**
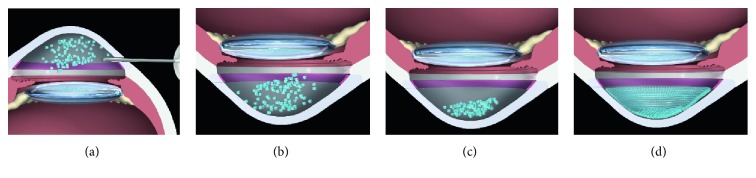
Schematic images of the cultured corneal endothelial cell (CEC) injection therapy. (a) Coinjection of cultured CECs with a ROCK inhibitor into the anterior chamber; (b) patient in the facedown position, to allow the CECs to sink down to the anterior chamber side of the cornea; (c) maintenance of the facedown position for 3 hours; (d) regeneration of the corneal endothelium by the injected cultured CECs. Reproduced from Okumura et al. [48].

In 2013, we obtained the approval from the Japanese Ministry of Health, Labour, and Welfare to initiate a first-in-man clinical trial of cell-based therapy to treat corneal endothelial dysfunction at the Kyoto Prefectural University of Medicine (Clinical trial registration: UMIN000012534) (Figure 5). Clinical data are still under analysis, but our initial clinical results seem to indicate that this treatment is both safe and effective. Further clinical data are anticipated that will determine the effectiveness in terms of clinical outcomes, such as visual acuity, CEC density, and rejection rate, when compared to conventional corneal transplantations.

**Figure 6:**
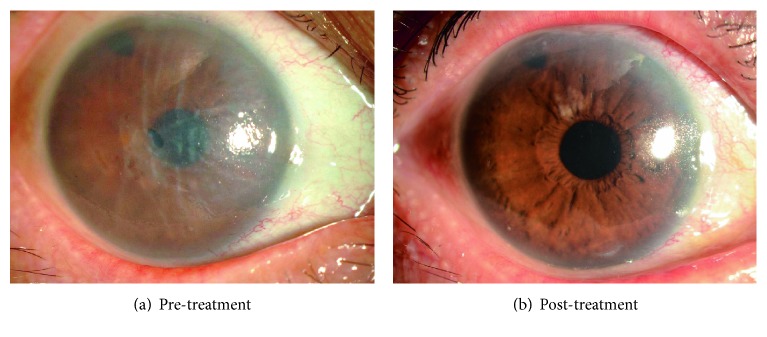
Representative images of the effectiveness of cultured corneal endothelial cell (CEC) injection therapy in clinical research. Representative slit-lamp microscopy images of the first patient, a Japanese female with corneal endothelial decompensation induced by argon laser iridotomy (a). After mechanical removal of an 8 mm diameter section of the corneal endothelium, cultured human CECs together with a ROCK inhibitor were injected into the anterior chamber. Preoperative visual acuity was 0.04 due to the edema in the corneal epithelium and stroma. The postoperative visual acuity recovered to 1.0, together with the associated recovery of corneal transparency (b).

Representative slit-lamp microscopy images from our first patient, a Japanese female with corneal endothelial decompensation induced by argon laser iridotomy, are shown in Figure 6. We mechanically removed an area approximately 8 mm diameter from the corneal endothelium, without removing Descemet's membrane, and injected cultured human CECs in combination with a ROCK inhibitor into the anterior chamber. The patient was kept in a facedown position overnight to enable the injected CECs to settle onto the Descemet's membrane. The patient's preoperative visual acuity was 0.04 and she had corneal epithelial and stromal edema, whereas her postoperative visual acuity recovered to 1.0 and was associated with the recovery of corneal transparency (Figure 6).

## 9. Conclusions

We have demonstrated that ROCK inhibitors, supplied in the form of eye drops, promote cell proliferation in animal models and most likely in humans. However, the development of these drugs to target corneal endothelium damage will require randomized clinical trials of (1) ROCK inhibitor eye drop administration following central corneal endothelial removal, either by Descemet's membrane removal or cryoprobe treatment, for the treatment of Fuchs endothelial corneal dystrophy and (2) ROCK inhibitor eye drop administration for the treatment of acute corneal endothelial damage due to cataract surgery. The use of ROCK inhibitors in tissue engineering therapy is also very promising, as indicated by the enhanced engraftment of CECs onto recipient tissues. We have treated 31 patients with cell injection therapy at the time of this review article. The collected data are currently undergoing an independent review to ensure diligent assessment of our clinical results for evaluation of the safety and effectiveness of this treatment.

For decades, the only therapy for the corneal endothelium has been corneal transplantation. Therefore, the promising responses to ROCK inhibitors would appear to open up new therapeutic possibilities.
